# Efficacy and Safety of Biodegradable Polymer Biolimus-Eluting Stents versus Durable Polymer Drug-Eluting Stents: A Meta-Analysis

**DOI:** 10.1371/journal.pone.0078667

**Published:** 2013-11-11

**Authors:** Yicong Ye, Hongzhi Xie, Yong Zeng, Xiliang Zhao, Zhuang Tian, Shuyang Zhang

**Affiliations:** 1 Department of Cardiology, Peking Union Medical College Hospital, Peking Union Medical College & Chinese Academy of Medical Sciences, Beijing, China; Cliniche Humanitas Gavazzeni, Italy

## Abstract

**Backgrounds:**

Drug-eluting stents (DES) with biodegradable polymers have been developed to address the risk of thrombosis associated with first-generation DES. We aimed to determine the efficacy and safety of biodegradable polymer biolimus-eluting stents (BES) versus durable polymer DES.

**Methods:**

Systematic database searches of MEDLINE (1950 to June 2013), EMBASE (1966 to June 2013), the Cochrane Central Register of Controlled Trials (Issue 6 of 12, June 2013), and a review of related literature were conducted. All randomized controlled trials comparing biodegradable polymer BES versus durable polymer DES were included.

**Results:**

Eight randomized controlled trials investigating 11,015 patients undergoing percutaneous coronary interventions were included in the meta-analysis. The risk of major adverse cardiac events did not differ significantly between the patients treated with the biodegradable polymer BES and the durable polymer DES (Relative risk [RR], 0.970; 95% CI, 0.848–1.111; p = 0.662). However, biodegradable polymer BES was associated with reduced risk of very late ST compared with the durable polymer DES, while the risk of early or late ST was similar (RR for early or late ST, 1.167; 95% CI 0.755–1.802; p = 0.487; RR 0.273; 95% CI 0.115–0.652; p = 0.003; p for interaction = 0.003).

**Conclusions:**

In this meta-analysis of randomized controlled trials, treatments with biodegradable polymer BES did not significantly reduce the risk of major adverse cardiac events, but demonstrated a significantly lower risk of very late ST when compared to durable polymer DES. This conclusion requires confirmation by further studies with long-term follow-up.

**PROSPERO register number:**

http://www.crd.york.ac.uk/PROSPERO/display_record.asp?ID=CRD42013004364#.UnM2lfmsj6J

## Introduction

First-generation drug-eluting stents (DES) made of durable polymer are well-established for reducing the risk of restenosis and the need for repeat revascularization than bare metal stents (BMS) [1], [2]. However, DES are associated with an increased risk of very late (>1 year) stent thrombosis (ST) as compared with BMS [3], [4]. The durable polymer in these DES can cause hypersensitivity reactions and chronic inflammation in the vessel wall persistently after completed drug release, and thus, may lead to delayed artery healing, incomplete re-endothelialization and artery remodeling, and very late stent thrombosis [5]–[7].

In order to overcome this issue, new DES platforms with biodegradable polymers have been developed [8] and early studies have demonstrated the these stents are feasible, safe, and effective both in short-term and long-term follow-up studies [9], [10]. Moreover, the polymer degradation kinetics have been well investigated both *in vitro* and *in vivo*, making it a potential new DES platform [11].

Biolimus (also known as umirolimus) is a highly lipophilic sirolimus derivative that, like sirolimus, is an inhibitor of cell growth, most likely via binding to the FK-binding protein and subsequently inhibiting mammalian target of rapamycin (mTOR) [12]. The affinity of biolimus A9 to FK-binding protein and its potency for inhibiting the proliferation of lymphocytes and coronary smooth muscle cells, as tested and demonstrated in several different *in vitro* models, are within the same order of magnitude as those of sirolimus, everolimus, and zotarolimus [13]. Biolimus-eluting stents (BES) were designed with a biodegradable polymer applied to the non-luminal surface of the stent. After implantation, the polymer is metabolized to water and carbon dioxide within nine months [14]. Thus, biolimus is released gradually through biodegradable polymers, and the polymer will be absent from the vessel wall, thus decreasing the chance of persistent inflammation and thrombosis [15].

The potential clinical benefit of using biodegradable polymer BES has been under investigation by several large randomized controlled trials (RCTs). Recently, the results of two large RCTs comparing biodegradable polymer BES with durable polymer DES have been published simultaneously and concluded that biodegradable polymer BES ware at least not inferior to durable polymer DES in short-term follow-up studies [16], [17]. Meanwhile, the long-term follow-up results of previous RCTs, such as the LEADERS trial [18], are also available. Whether biodegradable polymer BES is better than durable polymer DES in terms of reduced risk of cardiovascular events or stent thrombosis (ST) is still under debate. Thus, we conducted a meta-analysis of RCTs to compare the efficacy and safety of biodegradable polymer BES with durable polymer DES in patients undergoing percutaneous coronary interventions.

## Methods

We designed a protocol that detailed the objective of our analysis, criteria for study inclusion/exclusion, assessment of study quality, primary outcome, and statistical methods in accordance with the PRISMA (Preferred Reporting Items for Systematic Reviews and Meta-Analyses) statement [19]. All the analyses were pre-specified and this study has been registered in PROSPERO (international prospective registration of systematic reviews; Registration number: CRD42013004364; http://www.crd.york.ac.uk/PROSPERO/display_record.asp?ID=CRD42013004364).

### Data Sources and Searches

We conducted a search of MEDLINE (1950 to June 2013) and EMBASE (1966 to June 2013) via EMBASE.com, and the Cochrane Central Register of Controlled Trials (Issue 6 of 12, June 2013) to identify all published RCTs that compared biodegradable polymer BES with durable polymer DES. Internet-based sources of information on the results of clinical trials in cardiology (www.cardiosource.com/clinicaltrials, www.clinicaltrialresults.com, and www.pcronline.com) were also searched. Additionally, we performed a manual search of the literature using the references of the original manuscripts, reviews, and meta-analyses. No language restrictions were imposed. The keywords for searches were as follows: “biolimus”, “biolimus a9”, “umirolimus”, and “degradable polymer stent”.

### Study Selection

Two reviewers (Y. Y. and H. X.) independently determined the study eligibility. Any disagreements were resolved by consensus decisions. The eligibility criteria of the study characteristics included: (1) randomized controlled trials, (2) comparison of biodegradable polymer BES with durable polymer DES, and (3) reports of the major adverse cardiac events (MACE). Exclusion criteria included: (1) comparison of polymer free BES with durable polymer DES, and (2) the sample size of less than 50 patients.

### Data Extraction

Two authors (Y. Y. and H. X.) independently extracted the following data from the included studies. Any disagreements were resolved by discussion between the two reviewing authors. Information was extracted from each included trial on: (1) the study design; (2) characteristics of the participant (including number of participants, age, gender, and acute coronary syndrome); (3) past medical history (diabetes, hypertension, smoking, previous myocardial infarction [MI], previous coronary intervention, and previous coronary artery bypass surgery) (4) intervention (including types of stents, length of stents, number of stents, length of follow-up, and duration of dual antiplatelet therapy [DAPT]); and (5) clinical outcome (including MACE, definition of MACE, death, cardiac death, MI, target vessel revascularization [TVR], target lesion revascularization [TLR] and ST).

### Risk of Bias Assessment

Two authors (Y. Y. and H. X.) independently assessed the internal validity of the eligible studies according to the Cochrane Collaboration risk of bias tool. Disagreements were resolved in discussion with S. Z. until a consensus was obtained. The risk of bias was described and assessed in six specific domains: 1) sequence generation; 2) allocation concealment; 3) blinding of participants, personnel, and outcome assessors; 4) incomplete outcome data; 5) selective outcome reporting; and 6) other sources of bias. These judgments were based on the published study report that was included and based on a combination of study reports, protocols, and published comments regarding the study. The judgments involved used the answers “yes” (indicating a low risk of bias), “no” (indicating a high risk of bias), and “unclear” (if risk of bias is unknown or if an entry is not relevant to the study).

### Study Outcomes and Definitions

The primary outcome was MACE at the longest available follow-up. The definition of MACE in each individual study was accepted, although the definition of MACE differed between studies (Table 1). The secondary outcomes were the individual endpoints of death, cardiac death, MI, clinically indicated TVR, clinically indicated TLR, and definite or probable ST (including early, late, and very late ST) as defined according to the Academic Research Consortium (TRC) [20].

**Table 1:** Characteristics of included studies.

Studies	Year	Study population	Design	Age, year	Male,%	ACS,%	N	Durationof DAPT||,months	Follow-up,months	MACE^#^ definition
COMPARE II^16^	2013	Patients eligible for PCI*	open-label, randomized (2∶1),controlled, non-inferiority	62.9	74.4	57.9	2707	12	12	Cardiac death, MI**, and clinically indicated TVR††
SORT OUT V^17^	2013	SAP†/ACS‡+ at least one >50% stenosis	open-label, randomized (1∶1),controlled, non-inferiority	65.1	74.8	48.9	2468	12	12	Cardiac death, MI, definite ST‡‡, and clinically-driven TVR
NOBORI 1 (Phase 1+2)^25–26,31^	2007	SAP/UA§/provocable ischemia eligible for PCI	open-label, randomized (2∶1),controlled, non-inferiority	62.9	72.4	28.0	263	6	24	Cardiac death, MI, emergent cardiac bypass surgery, and TVR
LEADERS^18,27^	2008	SAP or ACS eligible for PCI	Single-blinded, randomized (1∶1),controlled, non-inferiority	64.6	74.8	55.2	1707	12	48	Cardiac death, MI, and clinically-indicated TVR
NOBORI JAPAN^28^	2011	SAP/UA/provocable ischemia eligible for PCI	open-label, randomized (3∶2),controlled	67.3	71.8	14.4	335	3	9	Cardiac death, MI, and TLR§§
Separham, et al^30^	2012	Patients eligible for PCI	open-label, randomized (1∶1),controlled	NA	NA	NA	200	NA	12	Cardiac death, MI, and TVR
NEXT^29^	2013	Patients eligible for PCI	Assessor-blinded, randomized(1∶1), controlled	69.2	77	16.5	3235	3	12	Cardiac death, MI, and TLR

* PCI: percutaneous coronary intervention;

† SAP: stable angina pectoris;

‡ ACS: acute coronary syndrome;

§UA: unstable angina;

|| DAPT: dual antiplatelet therapy (protocol mandated); ^#^MACE: major adverse cardiac events;

** MI: myocardial infarction;

†† TVR: target vessel revascularization;

‡‡ ST: stent thrombosis;

§§ TLR: target lesion revascularization.

### Data Synthesis and Analysis

The κ statistic was used to assess the agreement between reviewers for study selection. The pooled relative risk (RR) was calculated for each outcome using the inverse-variance method for random effect, as well as for fixed effects [21]. The data heterogeneity was assessed using the Cochrane Q test via a χ^2^ test and quantified with the I^2^ test [22].

Subgroup and meta-regression analyses were used to explore the potential sources of heterogeneity among the included studies. The following covariates were analyzed in subgroup analyses to explore the heterogeneity: 1) protocol-mandated DAPT duration: 12 months or less, 2) length of follow-up: more than 12 months or within 12 months, and 3) different types of durable polymer DES. Exploratory meta-regression was conducted to assess heterogeneity quantitatively among the studies. We used the log RR as the dependent variable. The log RR standard error was used to measure the within-study variability, and the residual maximum likelihood method was used to estimate the between-study variance. The mean age, proportion of males, diabetes and ACS in each study were included in the meta-regression model as covariates.

Sensitivity analysis was used to explore the degree to which the main findings of our meta-analysis were affected by individual studies. The publication bias was assessed by the Begg’s funnel plot and the Egger weighted regression statistic, where a value of *p*<0.10 indicates a significant publication bias among the included studies [23], [24].

All analyses were performed using STATA version 11.0 (Stata Corp; College Station, TX).

## Results

### Characteristics of Included Studies

**Figure 1:**
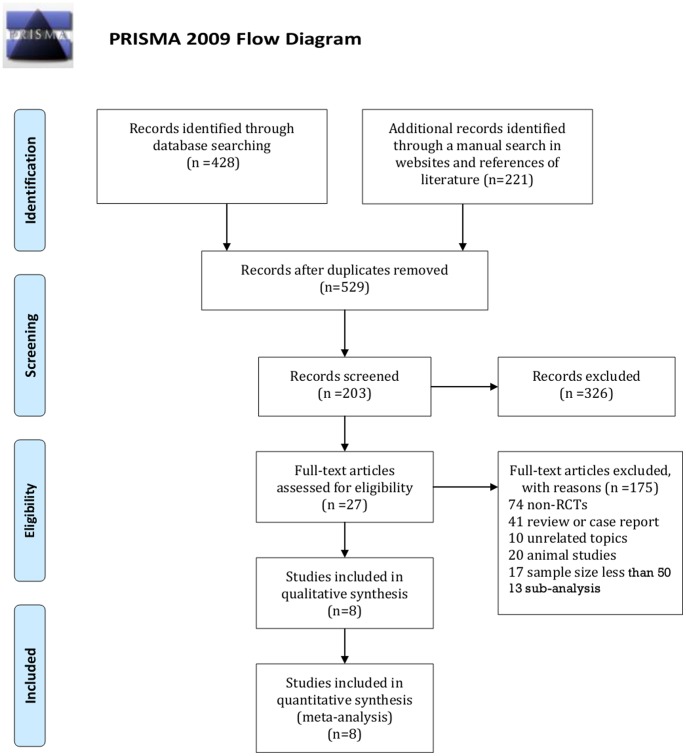
Flowchart for study selection.

Five-hundred and twenty-nine records were retrieved from the initial search. Twenty-seven studies were reviewed in full-text. Eight full-text articles [16]–[18], [25]–[29] and 2 meeting abstracts [30], [31] investigating 8 RCTs were included in the meta-analysis, one of which is only available in the form of a meeting abstract [30] (Fig. 1). The final 5 year follow-up of the LEADERS trial has been reported in TCT 2012 as an abstract [32]. However, the data were not sufficient for analysis in this final report, and therefore we used the 4 year follow-up data of the LEADERS trial in our meta-analysis [18]. Phase 1 and Phase 2 studies [26] of NOBORI 1 were presented as one study in our meta-analysis. The inter-reviewer agreement for the study selection was high with κ = 0.97.

The reported patients’ mean age ranged from 62.9 to 69.2 years, and each trial predominantly enrolled men. The characteristics of study population are presented in Table 1–3. Of the total 11,015 included patients, 6,034 were scheduled to receive biodegradable polymer BES and 4,981 to receive durable polymer DES. Three RCTs compared biodegradable polymer BES with durable polymer Sirolimus-eluting stents, three with durable polymer Everolimus-eluting stents, and two with durable polymer Paclitaxel-eluting stents. The longest available follow-up periods ranged from 9 to 48 months. The protocol’s mandatory DAPT durations for these trials were 3 months, 6 months, and 12 months.

**Table 2:** Characteristics of included studies.

Studies	DM*,%	HTN†,%	Currentsmoker,%	PreviousMI‡, %	PreviousPCI§, %	PreviousCABG||, %	Stent length(per patient),mm	Stent length(per lesion),mm	Stentnumbers(per patient),n	Stentnumbers(per lesion),n
COMPARE II^16^	21.7	55.3	29.7	19.8	17.5	5.8	NA	NA	NA	1.4
SORT OUT V^17^	15.2	56.4	33.3	17.5	16.9	7.0	22.5	15	NA	NA
NOBORI 1 (Phase1+2)^25–26,31^	18.5	63.3	21.0	22.6	20.6	3.7	NA	NA	NA	NA
LEADERS^18,27^	24.3	73.1	24.6	32.4	36.6	11.5	NA	23.7	NA	1.3
NOBORI JAPAN^28^	39.0	79.8	22.7	20.9	35.1	1.5	NA	NA	NA	NA
Separham, et al^30^	NA	NA	NA	NA	NA	NA	NA	NA	NA	NA
NEXT^29^	46	81.5	18.5	28.0	50.5	5.1	32.9	27.1	1.6	1.3

* DM: diabetes mellitus;

† HTN: hypertension;

‡ MI: myocardial infarction;

§ PCI: percutaneous coronary intervention;

|| CABG: coronary artery bypass.

**Table 3:** Clinical outcomes of included studies.

Studies	Intervention	N	MACE*	AllDeath	CardiacDeath	MI†	TVR‡	TLR§	Definite orprobableST||	Intervention	N	MACE	All Death	Cardiac Death	MI	TVR	TLR	Definite or probable ST
COMPARE II^16^	Biolimus	1795	93	27	14	51	52	37	14	Everolimus	912	44	10	7	23	24	16	9
SORT OUT V^17^	Biolimus	1229	66	30	12	19	52	40	10	Sirolimus	1239	55	27	14	11	39	25	4
NOBORI 1(Phase 1+2)^25–26,31^	Biolimus	238	19	/	/	/	/	/	0	Paclitaxel	125	11	/	/	/	/	/	6
LEADERS^18,27^	Biolimus	857	160	79	51	71	91	74	30	Sirolimus	850	192	87	57	73	110	93	39
NOBORI JAPAN^28^	Biolimus	198	10	2	1	8	5	1	0	Sirolimus	137	8	0	0	3	5	5	0
Separham, et al^30^	Biolimus	100	2	0	0	2	0	0	0	Everolimus	100	0	0	0	0	0	0	0
NEXT^29^	Biolimus	1617	116	41	25	53	105	47	4	Everolimus	1618	109	40	19	50	99	47	1

* MACE: major adverse cardiac events;

† MI: myocardial infarction;

‡ TVR: target vessel revascularization;

§ TLR: target lesion revascularization;

|| ST: stent thrombosis.

The definition of MACE in each of the trials represented combination outcomes of cardiac death, MI, and repeat revascularization (including TVR/TLR with or without emergent cardiac bypass surgery). A definite ST was included as a component of MACE only in SORT OUT V trials [17].

### Risk of Bias Assessment

All trials were reported in full-length articles except one trial from Separham, et al. [29], where the risk of bias is unclear. The remaining studies, which included COMPARE II [16], SOUT OUT V [17], LEADERS [18], [27], and NEXT [29] trials, are well-designed RCTs with pre-specified protocols, which reduces the risk of bias. The NOBORI 1 study [18], [19], [23] has no sufficient information for random sequence generation, lost to follow-up data, or a pre-specified protocol, and therefore has an unclear risk of selection bias, attrition bias, and reporting bias. NOBORI JAPAN [21] reported no information on random sequence generation, allocation concealment, and lacked a pre-specified protocol, and therefore has an unclear risk of selection bias and reporting bias. The results on the risk of bias assessment were summarized as a graph and are presented in figure S1.

### Quantitative Data Analysis

**Figure 2:**
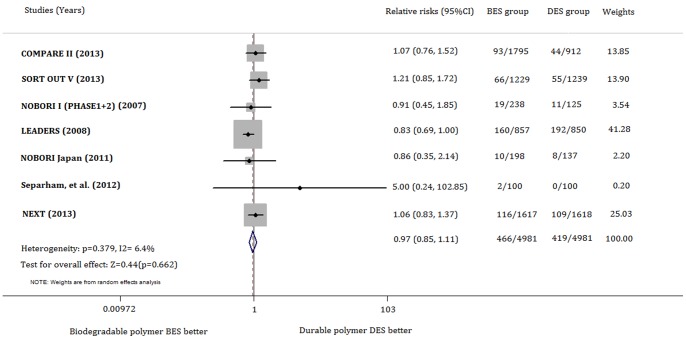
Forest plot for MACE associated with biodegradable polymer BES versus durable polymer DES. The squares and the horizontal lines indicate the relative risks, and the 95% confidence intervals for each trial included the size of each square is proportional to the statistical weight of a trial in the meta-analysis. A diamond indicates the effect estimate derived from meta-analysis, with the center indicating the point estimate, and the left and the right ends are the 95% CI. MACE: major adverse cardiac events; BES: biolimus-eluting stents; DES: drug-eluting stents.

The risk of MACE was not significantly different between the biodegradable polymer BES-treated and control groups either in a random-effect model (RR 0.970; 95% CI, 0.848–1.111; p = 0.662) or in a fixed-effect model (RR 0.962; 95% CI, 0.849–1.090; p = 0.540). No obvious heterogeneity was identified among the included studies (χ^2^ = 6.41, p for χ^2^ = 0.379; *I* ^2^ = 6.4%) (Fig. 2).

**Figure 3:**
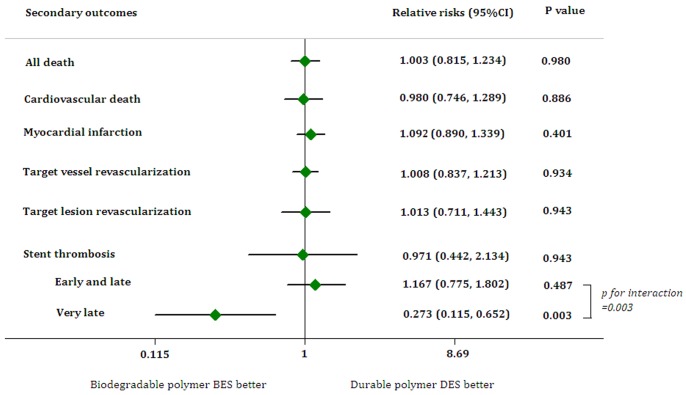
Forest plot for secondary outcomes associated with biodegradable polymer BES versus durable polymer DES. The squares and the horizontal lines indicate the relative risks, and the 95% confidence intervals for each secondary outcome. BES: biolimus-eluting stents; DES: drug-eluting stents.

The use of biodegradable polymer BES resulted in similar risk of all-cause death, cardiac death, or MI in both random effect and fixed effect model without heterogeneity (all-cause death: RR, 1.003; 95% CI, 0.815–1.234; p = 0.980; χ^2^ = 2.09, p for χ^2^ = 0.720, *I* ^2^ = 0%) (Cardiac death: RR 0.980; 95% CI, 0.746–1.289; p = 0.886; χ^2^ = 1.56, p for χ^2^ = 0.816, *I* ^2^ = 0%) (MI: RR 1.092; 95% CI, 0.890–1.339; p = 0.401; χ^2^ = 3.77, p for χ^2^ = 0.584, *I* ^2^ = 0%). The risk of clinically-indicated TVR in the biodegradable polymer BES group did not differ from the durable polymer DES group with moderate heterogeneity across studies (Random effect: RR 1.008; 95% CI, 0.837–1.213; p = 0.934; Fixed effect: RR 0.997; 95% CI, 0.851–1.168; p = 0.974; χ^2^ = 4.93, p for χ^2^ = 0.295, *I* ^2^ = 18.8%), as well as the risk of clinically-indicated TLR (Random effect: RR 1.013; 95% CI, 0.711–1.443, p = 0.943; Fixed effect: RR 0.969; 95% CI, 0.795–1.181; p = 0.756; χ^2^ = 9.66, p for χ^2^ = 0.047, *I* ^2^ = 58.6%). The pooled RR of definite or probable ST was similar for the biodegradable polymer BES group and the control group with significant heterogeneity (Random effect: RR 0.971; 95% CI, 0.442–2.134, p = 0.942; Fixed effect: RR 0.870; 95% CI, 0.598–1.266; p = 0.466; χ^2^ = 9.86, p for χ^2^ = 0.043, *I* ^2^ = 59.4%). Data from early or late ST were available in all 8 studies comparing biodegradable polymer BES with durable polymer DES. Only NOBORI 1 (phase 1), NOBORI 1 (phase 2), and the LEADERS trial have follow-ups for more than 12 months and thus have data for very late ST. The pooled risk of early- or late ST was identical between biodegradable polymer BES and durable polymer DES (RR 1.167; 95% CI 0.755–1.802; p = 0.487) (Heterogeneity: χ^2^ = 7.86, p for χ^2^ = 0.097, *I* ^2^ = 49.1%), while biodegradable polymer BES significantly reduced the risk of very late ST compared with durable polymer DES (RR 0.273; 95% CI 0.115–0.652; p = 0.003; p for interaction = 0.003) (Heterogeneity: χ^2^ = 0.41, p for χ^2^ = 0.52, *I* ^2^ = 0%) (Fig. 3).

**Figure 4:**
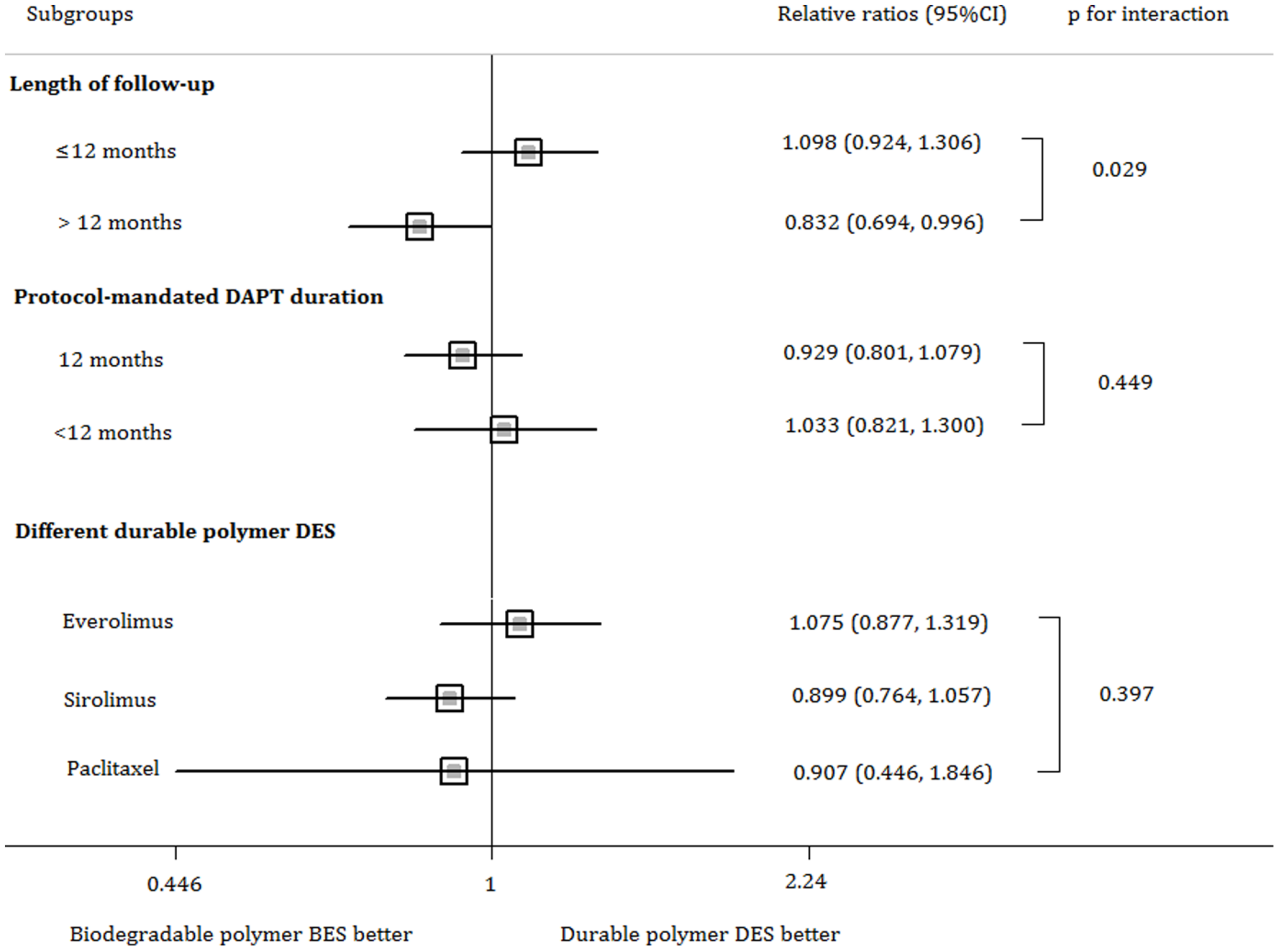
Subgroup analysis on MACE associated with biodegradable polymer BES versus durable polymer DES. The squares and the horizontal lines indicate the relative risks and the 95% confidence intervals for each subgroup. MACE: major adverse cardiac events; BES: biolimus-eluting stents; DES: drug-eluting stents.

The subgroup analyses showed that neither protocol-mandated DAPT duration nor different types of durable polymer DES significantly affected the RR of biodegradable polymer BES versus durable polymer DES. However, patients in the biodegradable polymer BES group who had a long-term follow-up (length of follow-up>12 months) were associated with a decreased risk of MACE. (Fig. 4). Meta-regression analyses showed no significant interactions between the log RR of MACE and mean age, proportion of males, diabetes, or ACS (Table S1).

The sensitivity of analysis found that the pooled RRs excluding each individual study were comparable, indicating the pooled RR was not affected by a single study (Table S2). Assessments of data using the Begg’s funnel plot (p = 0.881) and Egger’s weighted regression statistic (p = 0.253) indicated no significant publication bias.

## Discussion

In this meta-analysis of 8 RCTs, no significant difference was found in the patients’ risk of MACE between using the biodegradable polymer BES and the durable polymer SES, as well as other secondary outcomes, including all cause death, cardiac death, MI, clinically indicated TVR, clinically indicated TLR, and definite or probable ST. However, the use of biodegradable polymer BES resulted in a significantly lower risk of very late ST in patients when compared with durable polymer DES, while the risk of early or late ST between these was similar.

Biolimus, a new sirolimus derivative, is modified at position 40 of the rapamycin ring to enhance its lipophilicity and elution rate relative to the parent drug. This modification results in an increased uptake in local target tissues and therefore a reduced presence in systemic circulation. The NOBORI 1 clinical trial showed that the Biolimus A9-eluting stent is as effective as the Taxus stent in reducing neointimal proliferation [25]. However, the potential clinical advantage of a biodegradable polymer might be expected to emerge once the polymer has dissolved, and this may have occured 9 months after implantation [14], [25]; thus, it is not surprising that biodegradable BES did not reduce the risk of MACE in our meta-analysis, in which most of the studies only report endpoints within 12 months. Our subgroup analysis indicated a benefit towards the biodegradable polymer BES group with long-term follow-up. The potential long-term benefit of the biodegradable polymer BES still needs to be confirmed using the follow-up data of these studies.

Incomplete endothelialization of the stent struts and positive vessel remodeling due to chronic inflammation is believed to cause very late ST because the persistent polymer material on durable polymer DES after completed drug release might trigger an inflammatory response [5], [6]. Animal studies have shown significantly lower inflammatory responses in the stented segments, and the rapid recovery of endothelial function of peri-stent segments in the biodegradable polymer BES group compared with the durable polymer DES group at 1 month [33]. Also, the level of endothelial coverage in biodegradable polymer BES was comparable to BMS at four weeks, with no significant increase in inflammatory reactions up to 15 months [34]. These observations may explain why the biodegradable polymer BES may be associated with a lower risk of very late ST and a better long-term outcome in our meta-analysis.

The safety and efficacy of several other biodegradable polymer DES have also been investigated in recent RCTs, and the results are consistent with our meta-analysis. The ISAR-TEST 4 trial compared a biodegradable polymer sirolimus-eluting stent with the durable polymer sirolimus-eluting stent or the durable polymer everolimus-eluting stent in 2,603 patients, and we noted no difference between the biodegradable polymer stent and combined durable polymer stent groups in terms of a composite of cardiac death, target vessel-related MI, and TLR [35]. The EVOLVE trial compared two doses of a biodegradable polymer everolimus-eluting stent with a durable polymer everolimus-eluting stent and demonstrated that both dose formulations of the biodegradable polymer stent were not inferior when compared with the durable polymer stent in terms of late in-stent loss at 6 months [36]. Recently, a pooled analysis of individual patient data from the ISAR-TEST 3, ISAR-TEST 4, and LEADERS trials by Stefanini et al. confirmed that the biodegradable polymer DES (BES or sirolimus-eluting stent) was associated with a significantly reduced risk of stent thrombosis, which was driven by a lower risk of very late stent thrombosis (hazard ratio 0.22, 95% CI 0.08–0.61, P = 0.004) [37]. They also found that the risk of TLR was significantly lower among patients treated with biodegradable polymer DES compared to durable polymer SES (hazard ratio 0.82, 95% CI 0.68–0.98, P = 0.029) [37], while there was no significant difference in TLR in our meta-analysis. The major reasons for this difference are as follows: first, our studies included eight studies involving only biodegradable polymer BES, while Stefanini et al. included three studies with two different types of biodegradable polymer DES (one studies with BES, two with sirolimus-eluting stents). Second, all of three studies had long-term outcomes (4 years), while most of our included studies only had short-term follow-up data available. Thus, whether biodegradable polymer BES is associated with a low risk of TLR needs to be verified by long-term follow-up studies.

Our meta-analysis study has some limitations. First, this is a study level meta-analysis instead of an individual level meta-analysis, which makes it impossible to investigate the role of several confounding factors at the patient level. Second, we grouped all types of durable polymer DES as a control group, since there may be potential heterogeneity across different DES. However, our subgroup analysis indicated no significant interactions among different types of durable polymer DES. Third, the results of the subgroup analysis may be limited by the small number of studies in each subgroup, and a moderate degree of heterogeneity in some secondary outcome analyses may lessen the reliability of the conclusion. Finally, although the analyses identified statistically insignificant publication bias, the Begg’s or Egger’s tests may be underpowered when the number of studies is small. Moreover, there may be data relevant to this topic that has not been published.

Based on the current available evidence, the biodegradable polymer BES did not significantly improve clinical outcomes when compared with the durable polymer DES, with the exception of the risk for very late ST. Long-term follow-up data are required before we can make recommendations for the role of biodegradable polymer BES use in routine clinical practice.

## Supporting Information

Figure S1 **Risk of bias assessment.**
(TIF)

Table S1 **Results of meta-regression.**
(DOC)

Table S2 **Sensitivity analysis of the effect of exclusion of individual studies on pooled relative risks (RRs) with 95% confidence intervals (CI).**
(DOC)

Checklist S1 (DOC)
