# Supplementary figures and images for: Efficacy and Safety of Biodegradable Polymer Biolimus-Eluting Stents versus Durable Polymer Drug-Eluting Stents: A Meta-Analysis
Source: PLoS One. 2013 Nov 11;8(11):e78667. doi: 10.1371/journal.pone.0078667 (PMC3823917; doi:10.1371/journal.pone.0078667)

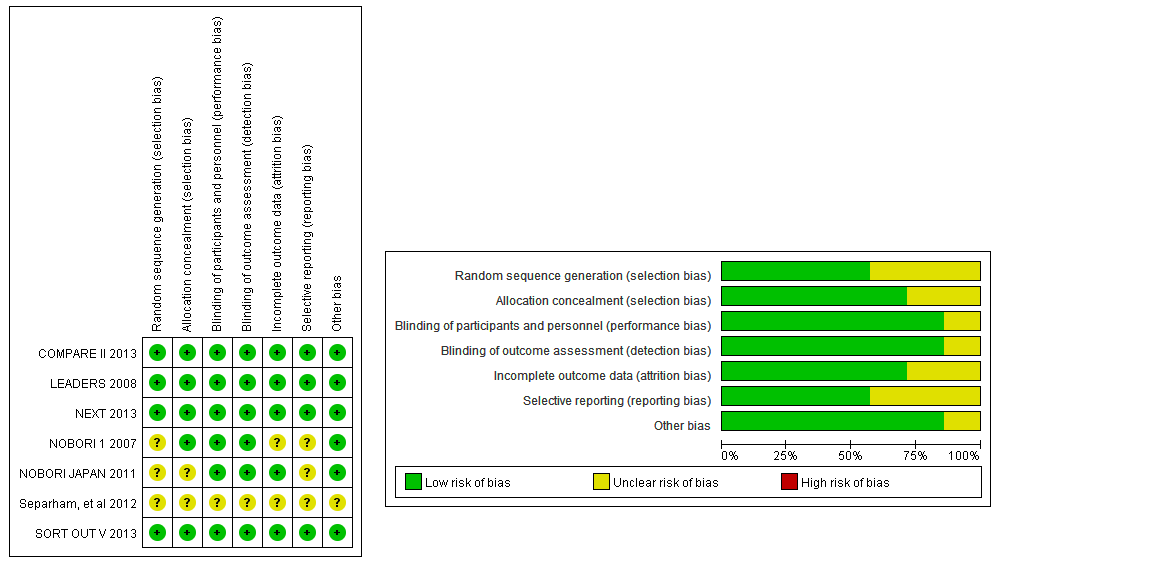

Supplement: Figure S1 — Risk of bias assessment. (TIF) [file pone.0078667.s001.tif]
