# Supplementary material for: Efficacy and Safety of Biodegradable Polymer Biolimus-Eluting Stents versus Durable Polymer Drug-Eluting Stents: A Meta-Analysis
Source: PLoS One. 2013 Nov 11;8(11):e78667. doi: 10.1371/journal.pone.0078667 (PMC3823917; doi:10.1371/journal.pone.0078667)
Supplement: Table S1 — Results of meta-regression. (DOC) [file pone.0078667.s002.doc]

Table S1. Results of meta-regression

| Variants | Coefficients | 95% confidence intervals | p value |
| --- | --- | --- | --- |
| Mean Age | 0.009 | -0.065~0.082 | 0.753 |
| Male | 1.847 | -12.952~16.645 | 0.746 |
| Diabetes | 0.086 | -1.552~1.724 | 0.891 |
| Acute coronary syndrome | -0.150 | -0.118~0.882 | 0.707 |
