# Supplementary material for: Efficacy and Safety of Biodegradable Polymer Biolimus-Eluting Stents versus Durable Polymer Drug-Eluting Stents: A Meta-Analysis
Source: PLoS One. 2013 Nov 11;8(11):e78667. doi: 10.1371/journal.pone.0078667 (PMC3823917; doi:10.1371/journal.pone.0078667)
Supplement: Table S2 — Sensitivity analysis of the effect of exclusion of individual studies on pooled relative risks (RRs) with 95% confidence intervals (CI). (DOC) [file pone.0078667.s003.doc]

| Studies excluded | Pooled RRs of Remaining Studies (95% CI) | *p* for RR |
| --- | --- | --- |
| COMPARE II, 2013 | 0.966 (0.820-1.139) | 0.683 |
| SORT OUT V, 2013 | 0.930 (0.813-1.063) | 0.286 |
| NOBORI 1 (Phase 1+2), 2007 | 0.988 (0.843-1.159) | 0.883 |
| LEADERS, 2008 | 1.087 (0.918-1.285) | 0.333 |
| NOBORI JAPAN, 2011 | 0.987 (0.844-1.155) | 0.874 |
| Separham, et al, 2012 | 0.965 (0.846-1.100) | 0.592 |
| NEXT, 2013 | 0.951 (0.803-1.125) | 0.555 |

Table S2. Sensitivity analysis of the effect of exclusion of individual studies on pooled relative risks (RRs) with 95% confidence intervals (CI)
